# Nanovolume optimization of protein crystal growth using the microcapillary protein crystallization system

**DOI:** 10.1107/S0021889810027378

**Published:** 2010-08-19

**Authors:** Cory J. Gerdts, Glenn L. Stahl, Alberto Napuli, Bart Staker, Jan Abendroth, Thomas E. Edwards, Peter Myler, Wesley Van Voorhis, Peter Nollert, Lance J. Stewart

**Affiliations:** a Emerald BioStructures Inc., 7869 NE Day Road West, Bainbridge Island, WA 98110, USA; b Emerald BioSystems Inc., 7869 NE Day Road West, Bainbridge Island, WA 98110, USA; c Accelerated Technologies Center for Gene to 3D Structure, USA; d Seattle Structural Genomics Center for Infectious Disease, USA; e University of Washington, Seattle, WA 98195, USA; f Seattle BioMed, 307 Westlake Avenue North, Suite 500, Seattle, WA 98109, USA

**Keywords:** protein crystals, microfluids, plugs, genomics

## Abstract

The Microcapillary Protein Crystallization System (MPCS) is a microfluidic, plug-based crystallization technology that generates X-ray diffraction-ready protein crystals in nanolitre volumes. In this study, 28 out of 29 (93%) proteins crystallized by traditional vapor diffusion experiments were successfully crystallized by chemical gradient optimization experiments using the MPCS technology. In total, 90 out of 120 (75%) protein/precipitant combinations leading to initial crystal hits from vapor diffusion experiments were successfully crystallized using MPCS technology. Many of the resulting crystals produced high-quality X-ray diffraction data, and six novel protein structures that were derived from crystals harvested from MPCS CrystalCards are reported.

## Abstract 2

The Microcapillary Protein Crystallization System (MPCS) is used to successfully optimize protein crystals from 28 out of 29 tested proteins. Six protein structures have been determined from diffraction-ready crystals grown inside and harvested directly from the MPCS CrystalCards, which are compatible with the recently commercialized and automated MPCS Plug Maker instrument.

## 1. Introduction

New technologies to improve protein crystallization success rates are the focus of continuous research and technology development (Fox *et al.*, 2008 ▶; Ng, Clark *et al.*, 2008 ▶; Ng, Stevens & Kuhn, 2008 ▶; Li *et al.*, 2009 ▶, 2010 ▶; Hansen *et al.*, 2002 ▶; Cherezov *et al.*, 2008 ▶, 2009 ▶; Dhouib *et al.*, 2009 ▶; Sauter *et al.*, 2007 ▶; Hansen & Quake, 2003 ▶). Protein crystals are often so difficult to produce that crystallographers are willing to try a new crystallization technology, even if it might provide only a small chance at crystallization success. However, a new technology will only be widely accepted if it is able to demonstrate clear value to crystallographers. In this field, value to crystallographers is measured by the ease with which diffraction quality crystals and crystal structures can be produced from a limited amount of protein supply.

The Accelerated Technologies Center for Gene to 3D Structures (ATCG3D) has developed the Microcapillary Protein Crystallization System (MPCS), which is a plug-based, microfluidic protein crystallization technology capable of quickly and easily setting up hundreds of batch-under-oil-style crystallization experiments (Gerdts *et al.*, 2008 ▶). The MPCS is unique because it is capable of generating hundreds of nanovolume (10–20 nl) experiments, each containing a slightly different chemical composition (Gerdts *et al.*, 2006 ▶; Zheng *et al.*, 2003 ▶, 2005 ▶). The result is on-chip formulation of finely controlled concentration gradients over a series of drops (plugs) that are effective at optimizing protein crystals. Further, the peel-apart CrystalCards used as a part of the MPCS allow simple crystal extraction for diffraction studies. Combining these benefits yields a technology that is able to carefully optimize crystal hits, generating protein crystals that are ready for subsequent diffraction experiments. In this report we have examined the ability of the MPCS technology to perform crystal optimizations of 29 different soluble proteins provided by the Seattle Structural Genomics Center for Infectious Disease (SSGCID).

## 2. Research study workflow

**Figure 1:**
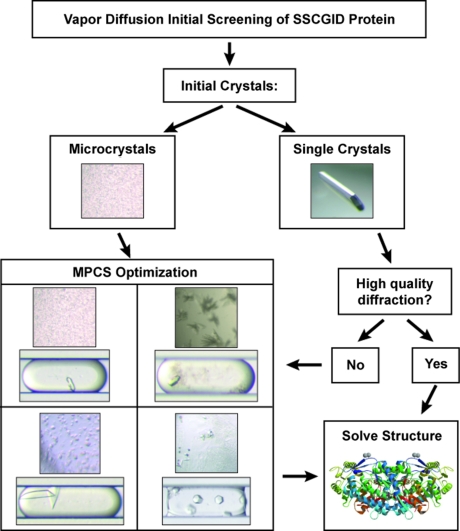
A flow chart describing the sequence of events undertaken in this study. Purified protein was received and initial screening *via* sitting-drop vapor-diffusion experiments was set up. If initial crystals were single and harvestable, they were analyzed *via* X-ray diffraction. If the initial protein crystals produced high-quality X-ray diffraction data, the structure was solved without MPCS optimization. However, if the initial X-ray diffraction data were poor, or if the initial crystals were small or not harvestable, the crystals were optimized using the MPCS.

SSGCID is one of two centers funded by the National Institute of Allergy and Infectious Diseases (NIAID) and is a consortium of four Pacific Northwest institutions (Seattle BioMed, Emerald, University of Washington and Battelle). SSGCID’s primary mission is to determine 75–100 new protein structures annually for targets from NIAID category A–C agents, as well as emerging and re-emerging infectious disease organisms for a period of five years. In this study, SSGCID proteins were used to test the ability of the MPCS technology to rapidly optimize protein crystallization conditions using crystallization hits from traditional sitting-drop vapor-diffusion crystallization trials. A chart describing the workflow of the study is shown in Fig. 1 ▶. Purified SSGCID proteins were screened – using sitting-drop vapor diffusion – against a series of common crystallization screens (Wizard I, Wizard II, Wizard III, JCSG+ and Precipitant Synergy from Emerald BioSystems, and Crystal Screen HT and Index HT from Hampton Research). Proteins that did not yield initial crystals were retired – consistent with the workflow of the high-throughput SSGCID structure determination pipeline. If the initial screens yielded single crystals ready for analysis by X-ray diffraction, they were first tested for diffraction quality before undergoing optimization using the MPCS (this was done to avoid optimizing crystals that were not in need of any improvement). Proteins that led to initial microcrystals or single crystals that did not produce high-quality X-ray diffraction underwent optimization using the MPCS. Thus, the proteins examined in this study were those that generated initial crystal hits but were otherwise randomly selected. In total, 29 proteins underwent MPCS optimizations.

**Figure 2:**
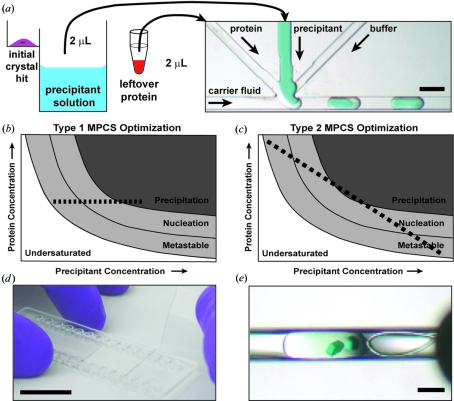
(*a*) *Ca* 2 µl of leftover protein solution and *ca* 2 µl of precipitant solution from the initial experiment (left) were used to generate an optimization experiment in the MPCS CrystalCard (right). In the CrystalCard, aqueous solutions (protein, precipitant and buffer) were combined and spontaneously segmented into individual drops (plugs) by the inert, immiscible carrier fluid. The resulting plugs filled the microcapillary and were incubated as individual crystallization experiments. Scale bar = 400 µm. (*b*), (*c*) Generic protein crystallization phase diagrams indicating how crystallization phase space is interrogated in MPCS optimizations. In Type 1 MPCS optimizations (*b*) protein concentration is held constant while a gradient of precipitant concentration is generated over a series of plugs. In Type 2 MPCS optimizations (*c*), protein concentration begins high and slowly decreases as precipitant concentration begins low and slowly increases to generate a dynamic protein *versus* precipitant gradient over a series of plugs. (*d*) A picture of an MPCS CrystalCard being peeled apart in order to expose the crystals. Scale bar = 1 inch ≃ 2.54 cm. (*e*) A picture of a protein crystal being harvested from a CrystalCard using a 0.2 mm cryo-loop. Scale bar = 200 µm.

MPCS optimization experiments generated highly granular gradients containing up to 400 individual crystallization experiments in 20 nl drops called plugs. Approximately 2 µl of leftover protein and 2 µl of the precipitant solution (used in the initial screen) were combined inside the microfluidic circuitry of the MPCS CrystalCard (Fig. 2 ▶ *a*). Each plug was formed at a slightly different concentration than the plugs before and after. This was accomplished by dynamically controlling the flow rates of the solutions used to form the plugs. Computer control of flow rates generated a wide variety of potential gradients. For simplicity, only two types of gradients were used in this study. The two types are shown schematically in Figs. 2 ▶(*b*) and 2 ▶(*c*). The goal of the two MPCS optimization types was to carefully interrogate a narrow region of crystallization phase space surrounding the initial hit. Type 1 optimizations maintained protein concentration in all of the plugs while varying the precipitant concentration. Type 2 optimizations varied the protein and precipitant concentration against one another in order to interrogate the effect of varied ratios of protein and precipitant. Completed optimization experiments were incubated in the CrystalCard at 100% humidity to allow for crystal growth. Crystals in plugs stored in CrystalCards at 100% humidity have been shown to be stable for more than six months. Additionally – although not pursued in this study – plugs can be intentionally dehydrated while in the CrystalCard to initiate crystal growth by controlling the humidity of the storage container. After crystals grew, they were harvested for analysis by X-ray diffraction by peeling back the thin plastic bonding layer (Fig. 2 ▶ *d*) and harvesting the protein crystal directly from the microcapillary (Fig. 2 ▶ *e*).

## 3. Materials and methods

Plastic CrystalCards were manufactured from cyclic olefin copolymer. Each CrystalCard has two separate microcapillaries with approximately 10 µl of useful volume. One optimization experiment may be performed in each microcapillary. Plug formation in the CrystalCard requires a low-surface-energy (hydrophobic) surface. This ensures that the carrier fluid (FC-40) preferentially wets the walls of the microcapillary. To prepare the microcapillary surface for plug formation, Cytonix PFC 502AFA solution is used to coat the inside of the microcapillary. To apply the coating, the CrystalCard is filled from the outlet with Cytonix 502AFA solution and incubated under ambient conditions for 0.5–1 h. The 502AFA solution is removed from the CrystalCard *via* vacuum, followed by curing at 333–343 K for 1 h.

The CrystalCard has four inlet ports for introducing liquids, one each for the carrier fluid (1), protein (2), precipitant (3) and buffer (4). The buffer, protein and precipitant inlet channels merge at the 3 + 1 mixer, where the aqueous solutions are combined and segmented into individual plugs by the inert and immiscible carrier fluid. Syringes and Teflon tubings are back-filled with the carrier fluid, and the desired amounts of the aqueous solutions are aspirated into the ends of the Teflon tubings. Connection to the CrystalCard is achieved *via* the Teflon tubing and a polypropylene connector that forms an airtight seal to the port in the CrystalCard. The component liquids of the experiment are placed in the Teflon tubing of the syringe pumping system and delivered to the CrystalCard in a manner described previously using the MicroPlugger pump-control software (Gerdts *et al.*, 2008 ▶). The positioning of the fluid lines on the CrystalCard is noted in Fig. 2 ▶.

**Figure 3:**
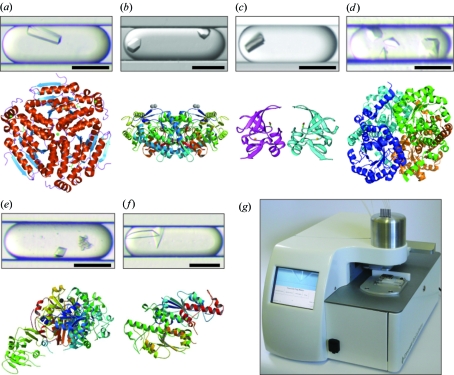
Pictures of crystals in plugs generated from MPCS optimizations that led to high-quality data sets (2.5 Å or better) and/or novel structures. Corresponding ribbon structures are included below the pictures of the plugs (for data collection and refinement statistics, see Table 2 ▶). All scale bars = 200 µm. (*a*) Enoyl-CoA hydratase from *Mycobacterium tuberculosis* (1.8 Å; PDB code 3h81); (*b*) aldehyde dehydrogenase from *Bartonella henselae* (2.1 Å; PDB code 3i44; deposited structure for PDB code 3i44 came from a sitting-drop optimization at 2.0 Å resolution; the 2.1 Å-resolution data set was generated from a crystal optimized using the MPCS); (*c*) methionine-*R*-sulfoxide reductase from *Burkholderia pseudomallei* (1.7 Å; PDB code 3cxk); (*d*) methylisocitrate lyase from *Brucella melitensis* (2.9 Å; PDB code 3eoo); (*e*) dihydrofolate reductase/thymidylate synthase from *Babesia bovis* (2.5 Å; PDB code 3i3r); (*f*) tRNA guanine-*n*1-methyltransferase from *Bartonella henselae* (2.5 Å; PDB code 3ief); (*g*) A picture of the commercial version of the MPCS Plug Maker. Left: The touch screen user interface and live image of the CrystalCard. Right: Instrument stage that holds the CrystalCard and crystallization samples.

The flow rates of the aqueous solutions can be varied such that a smooth gradient over a series of plugs is generated. In this study, we primarily used two types of gradients (Table 1 ▶ and Fig. 3 ▶). In gradient Type 1, the protein is delivered at a constant rate (2 µl min^−1^) while a linear gradient is made from precipitant and buffer solutions (with the sum of the precipitant and buffer flow rates remaining constant at 2 µl min^−1^). For most Type 1 gradients, the flow rate of the precipitant was programmed to start at 2 µl min^−1^ and slowly decrease as the flow rate of the buffer slowly increased at the same rate. Therefore in all Type 1 experiments, the concentration of the protein remains constant as the concentration of the precipitant varies (Table 1 ▶). In gradient Type 2, a dynamic gradient between the protein and precipitant is generated. In Type 2 gradients, the flow rate of the protein is slowly decreased as the flow rate of the precipitant is slowly increased and the buffer flow rate is held constant (Table 1 ▶). In general, a Type 1 gradient was generated for a protein/precipitant combination first and if, no crystals were seen, a Type 2 gradient was often generated as a follow-up.

Precipitants used for this study were available commercially from either Emerald Biosystems (Wizard I, Wizard II, Wizard III, JCSG+ and Precipitant Synergy) or Hampton Research (Crystal Screen HT and Index HT). Customized versions of Wizard I and Wizard II (also commercially available from Emerald BioSystems on request) were also used. The customized screen consisted of Wizard I and Wizard II with primary precipitant concentration increased by 50–100%.

Crystals were extracted directly from the CrystalCards for subsequent analysis by X-ray diffraction (Fig. 2 ▶ *d*). The 100 µm-thick plastic bonding layer was pealed off in order to expose the desired crystals to be harvested. Typically, *ca* 1 µl of a previously prepared cryo-protectant solution was pipetted directly onto the desired crystal. The crystal was then pulled out of the microcapillary using a traditional nylon cryo-loop (*ca* 0.2 mm diameter from Hampton Research) and stored in liquid nitrogen for transport to an X-ray source for analysis. On rare occasions, a crystal was found to remain on the 100 µm bonding layer. In this scenario, the crystal was still covered with the cryo-protectant solution and harvested from atop the plastic layer.

## 4. Results

MPCS gradients were shown to optimize vapor diffusion crystal hits with a high rate of success. Of the 29 proteins that underwent MPCS optimizations, 28 (93%) were crystallized using the MPCS. Many of the proteins used in this study produced crystals in more than one precipitant solution during the initial screening. In total, 120 different protein/precipitant combinations produced crystals in the vapor diffusion experiments. Of the 120 combinations, 90 (75%) produced crystals during the MPCS optimization experiments – a high success rate given that many of the initial crystals were not single crystals but tiny microcrystals or precipitation that looked crystalline (see examples in Fig. 1 ▶). In addition, MPCS experiments underwent a significant (50–100-fold) decrease in experimental volume and were translated from sitting-drop vapor-diffusion-style crystallization to batch-under-oil-style crystallization. Further, ten precipitant solutions that did not generate crystals in vapor diffusion experiments were used to generate crystals in MPCS optimization experiments. These particular precipitant solutions were tested using the MPCS (despite not yielding crystals from vapor diffusion experiments) because they possessed similarities in chemical composition to other precipitant solutions that did yield crystals in vapor diffusion experiments. In total, 17 precipitant solutions were tested in this manner and ten yielded crystals (59%). This indicates that using the MPCS to perform optimizations of every precipitant in the standard crystallization screens may generate many unique crystal hits that are being missed with a single vapor-diffusion experiment – consistent with previously reported data comparing vapor-diffusion-style crystallization with batch-under-oil-style crystallization (Baldock *et al.*, 1996 ▶; D’Arcy *et al.*, 2003 ▶). More than 90% of the precipitant solutions that were tested using vapor-diffusion experiments went untested in the MPCS, indicating a strong potential for discovering new crystal hits using the MPCS. A wide variety of solutions were represented in the precipitants used in this study, including various salt solutions, low- (4.5) to high-pH (10.5) solutions, high-viscosity polyethylene glycol solutions, and organics such as 2-propanol and 2-methyl-2,4-pentanediol.

## 5. Discussion

The goal of the MPCS optimizations was to salvage protein structures from initially screened proteins by (i) generating single crystals when sitting-drop experiments did not and/or (ii) improving the diffraction quality of initial single crystals generated in the sitting-drop experiments. Of the 29 protein targets involved in the study, six novel protein structures have been determined using crystals from MPCS optimizations and deposited in the PDB for a successful salvage rate of 21% (Figs. 3 ▶ *a*–3 ▶ *f*; for crystal optimization data, see Table 2 ▶). This salvage rate compares favorably to published data from reductive methylation (Kim *et al.*, 2008 ▶) and limited proteolysis (Dong *et al.*, 2007 ▶). In two cases, high-quality diffraction was also generated from crystals grown from subsequent sitting-drop vapor-diffusion experiments (in one case, X-ray diffraction from the MPCS crystal was of slightly higher resolution and in one case diffraction from the MPCS crystal was of slightly lower resolution). The development of the MPCS by ATCG3D has continued throughout this study, leading to the commercialization of the MCPS Plug Maker (Fig. 3 ▶ *g*). As shown in this study, the MPCS technology has been a successful method of optimizing protein crystals in order to yield high-quality X-ray diffraction results. Future directions for this technology are emerging and include incorporation of lipidic cubic phase into plugs for membrane protein crystallization (Li *et al.*, 2009 ▶) and high-throughput initial screening of protein samples with the hybrid method (Li *et al.*, 2006 ▶) (sparse matrix + gradient screening) made possible in an automated fashion by the availability of the MPCS Plug Maker.

## Figures and Tables

**Table 1:** Flow rate scheme (µl min^−1^) used for the Type 1 and Type 2 MPCS gradients in this study

		Protein	Precipitant	Buffer	Carrier fluid
Type 1	Starting flow rate	2	2		5
	Ending flow rate	2	0–1	1–2	5

Type 2	Starting flow rate	2		0.2	5
	Ending flow rate		2	0.2	5

**Table 2:** Crystal optimization data

Protein	Organism	Internal protein code	Potential crystal hits from initial screening	Precipitants tested using MPCS	Precipitants yielding crystals from MPCS	Best resolution from vapor diffusion crystals	Best resolution from MPCS crystals (Å)	Crystal structure?
Nucleoside diphosphate kinase	*Giardia lamblia*	Gila 438	23	23	16	–	6	No
Adenylate kinase	*Giardia lamblia*	Gila 297	10	10	8	–	8	No
Deoxynucleoside kinase	*Giardia lamblia*	Gila 1017	4	4	3	–	4	No
Arsenical pump-driving ATPase	*Giardia lamblia*	Gila 988	6	6	3	–	–	No
Peptide methionine sulfoxide reductase msrB	*Giardia lamblia*	Gila 536	1	1	1	–	–	No
Rab GDI	*Giardia lamblia*	Gila 634	2	2	2	–	–	No
Uracil phosphoribosyltransferase	*Giardia lamblia*	Gila 1401	2	2		–	–	No
Enoyl-CoA hydratase	*Mycobacterium tuberculosis*	Mytu 386	3	3	1	3.5	6	No
Enoyl-CoA hydratase	*Mycobacterium tuberculosis*	Mytu 358	76	8	6	1.8	2.05	Yes
Aldehyde dehydrogenase	*Bartonella henselae*	Bahe 886	79	7	7	2.0	2.0	Yes
Dihydrofolate reductase/thymidylate synthase	*Babesia bovis*	Babo 1191	18	10	6	–	2.5	Yes
Thymidylate synthase 1/2 TS-1	*Encephalitozoon cuniculi*	Encu 1191	2	2	2	3.1	None	No
Bifunctional dihydrofolate reductase-thymidylate synthase	*Toxoplasma gondii*	Togo 1191	3	3	2	–	3.8	No
Thymidylate synthase	*Burkholderia pseudomallei*	Bups 1181	3	3	1	–	–	No
UDP-*N*-acetylmuramate-L-alanine ligase	*Burkholderia pseudomallei*	Bups 137	5	5	4	6–7	6.8	No
RNA polymerase, α chain, bacterial and organelle	*Brucella melitensis*	Brab 66	3	3	1	–	none	No
tRNA (guanine-*n*1)-methyltransferase	*Bartonella henselae*	Bahe 1015	8	8	8	3	2.4	Yes
Endonuclease/exonuclease/phosphatase	*Giardia lamblia*	Gila 1102	2	2	1	None	None	No
Acetylglutamate kinase	*Bartonella henselae*	Bahe 993	8	4	4	5	4.5	No
Ribokinase	*Giardia lamblia*	Gila 1141	2	2	2	3.5	2.9	No
Probable thiosulfate sulfurtransferase	*Mycobacterium tuberculosis*	Mytu 1241	2	1	1	2.1	2.6	Yes†
Glycine cleavage system protein H	*Mycobacterium tuberculosis*	Mytu 1046	1	1	1	1.75	–	Yes†
Aldose reductase	*Giardia lamblia*	Gila 1452	2	1	1	2.7	3.6	No
Methionine-*R*-sulfoxide reductase	*Burkholderia pseudomallei*	Bups 33	2	2	2	–	1.7	Yes
Ribose-phosphate pyrophospho­kinase	*Burkholderia pseudomallei*	Bups 35	3	3	3	2.3	–	Yes†
δ-Aminolevulinic acid dehydratase	*Burkholderia pseudomallei*	Bups 75	1	1	1	–	–	No
Recombinase A	*Burkholderia pseudomallei*	Bups 69	1	1	1	–	–	No
Glutaryl-CoA dehydrogenase	*Burkholderia pseudomallei*	Bups 27	1	1	1	2.2	–	Yes†
Methylisocitrate lyase	*Burkholderia pseudomallei*	Bups 14	1	1	1	–	2.9	Yes

† The structure was solved after the completion of this study through subsequent salvage efforts.
